# Coexistence of *bla*_KPC_-IncFII plasmids and type I-E^*^ CRISPR-Cas systems in ST15 *Klebsiella pneumoniae*

**DOI:** 10.3389/fmicb.2023.1125531

**Published:** 2023-03-08

**Authors:** Yiyi Hu, Jianping Jiang, Dongliang Wang, Qinglan Guo, Minggui Wang

**Affiliations:** ^1^Institute of Antibiotics, Huashan Hospital, Fudan University, Shanghai, China; ^2^Key Laboratory of Clinical Pharmacology of Antibiotics, National Health Commission of People’s Republic of China, Shanghai, China; ^3^The First Department of Critical Care Medicine, Gansu Provincial Hospital, Gansu, China

**Keywords:** carbapenem-resistant *Klebsiella pneumoniae*, KPC-2, plasmid, CRISPR-Cas system, ST15

## Abstract

The CRISPR-Cas system in *Klebsiella pneumoniae* can prevent the entry of *bla*_KPC_-IncF plasmids. However, some clinical isolates bear the KPC-2 plasmids despite carrying the CRISPR-Cas system. The purpose of this study was to characterize the molecular features of these isolates. A total of 697 clinical *K. pneumoniae* isolates were collected from 11 hospitals in China, and tested for the presence of CRISPR-Cas systems using polymerase chain reaction. Overall, 164 (23.5%) of 697 *K. pneumoniae* isolates had type I-E^*^ (15.9%) or type I-E (7.7%) CRISPR-Cas systems. The most prevalent sequence type among isolates carrying type I-E^*^ CRISPR was ST23 (45.9%), followed by ST15 (18.9%). Isolates with CRISPR-Cas system were more susceptible to ten antimicrobials tested, including carbapenems, compared with the CRISPR-negative isolates. However, there were still 21 CRISPR-Cas-carrying isolates that showed resistance to carbapenems, and these isolates were subjected to whole-genome sequencing. Thirteen of these 21 isolates carried *bla*_KPC-2_-bearing plasmids, of which nine had a new plasmid type, IncFII_K34_, and two had IncFII(PHN7A8) plasmids. In addition, 12 of these 13 isolates belonged to ST15, while only eight (5.6%, 8/143) isolates belonged to ST15 in carbapenem-susceptible *K. pneumoniae* carrying CRISPR-Cas systems. In conclusion, we found that *bla*_KPC-2_-bearing IncFII plasmids could co-exist with the type I-E^*^ CRISPR-Cas systems in ST15 *K. pneumoniae*.

## Introduction

Prokaryotic organisms have developed numerous defense systems to protect themselves against parasitic nucleic acids, such as plasmids and viruses (bacteriophages; Hampton et al., 2020). Among these systems, clustered regularly interspaced short palindromic repeat (CRISPR) loci and CRISPR-associated (*cas*) genes dedicated to nucleic acid manipulation (Jansen et al., 2002) encode a unique defense mechanism that provides adaptive immunity against foreign elements. CRISPR loci are widely distributed and have been found in the genomes of about 42% of bacteria and 85% of archaea (Makarova et al., 2020). In these loci, an array of short, partially palindromic, repetitive noncoding DNA sequences is separated by equally short variable sequences known as spacers (Barrangou et al., 2007; Marraffini and Sontheimer, 2008; Pitout et al., 2015). Currently, CRISPRs are divided into two main classes, which encompass six major types (I–VI) and a total of 50 different subtypes based on their sequences (Makarova et al., 2020).

*Klebsiella pneumoniae* is an important human pathogen both in hospital and community settings. Increasing resistance to carbapenem in *K. pneumoniae* raises a global public health concern because of the prevalence of carbapenem-resistant *K. pneumoniae* (CRKP) and the associated high rate of mortality (Logan and Weinstein, 2017). The sequence types (STs) ST11 and ST258, belonging to clonal complex 258 (CC258), are well-established and compose the largest CRKP clonal group worldwide (Lee et al., 2016). Absence of the type I-E CRISPR-Cas system in *K. pneumoniae* CC258 was found to be associated with dissemination of IncF epidemic-resistance plasmids (Tang et al., 2020). *K. pneumoniae* ST15 is an emerging international high-risk clone causing nosocomial outbreaks worldwide (Lee et al., 2016) and the second most prevalent clone among CRKP isolates in China (Wang M. et al., 2022). Therefore, the molecular epidemiology of the CRISPR-Cas system of ST15 *K. pneumoniae* needs to be further determined.

In China, the dominant carbapenemase produced by CRKP is KPC-2, accounting for up to 94% (Wang M. et al., 2022). KPC-2 is generally located in the incompatibility group F (IncF) plasmids (Peirano et al., 2017). CRISPR-Cas systems identified in *K. pneumoniae* are categorized into type I-E and subtype I-E^*^ (Shen et al., 2017; Li et al., 2018). Previous studies have shown that the type I-E CRISPR-Cas system of *K. pneumoniae* can prevent the acquisition of the *bla*_KPC_-IncF plasmid (Zhou et al., 2020), and the type I-E^*^ CRISPR-Cas system of a *K. pneumoniae* isolate (NTUH-K2044) contributes to decrease of plasmid transformation and stability (Lin et al., 2016).

## Materials and methods

### Bacterial isolates

A total of 697 consecutive, non-duplicate clinical *K. pneumoniae* isolates were obtained from 11 hospitals in eight provinces in China between July 2018 and January 2019 (Supplementary Table S1). All of the isolates were initially identified as *K. pneumoniae* using a Vitek II system (bioMerieux, Marcy-l’Etoile, France) according to the manufacturer’s recommendations. *K. pneumoniae* was grown on Luria-Bertani (LB) agar or broth at 37°C. The isolates were stored at −80°C in LB broth containing 30% glycerol (v/v) until used.

### CRISPR-Cas system identification

DNA was extracted using the boiling method. The collected clinical isolates were tested for the presence of CRISPR-Cas systems (including type I-E^*^ and type I-E CRISPR systems) by polymerase chain reaction (PCR) using primers from a previous study (Li et al., 2018). PCRs were prepared using 2 × Hieff™ PCR Master Mix (with dye) (Yeasen) according to the manufacturer’s instructions (12.5 μL of Master Mix, 10 μM of each primer, and 1 μL DNA template, with a total volume of 25 μL).

### Multi-locus sequence typing

Multi-locus sequence typing (MLST) was performed on type I-E^*^ and type I-E CRISPR-containing isolates by amplifying seven housekeeping genes, *rpoB*, *gapA*, *mdh*, *pgi*, *phoE*, *infB*, and *tonB*. STs were assigned by querying the MLST database of *K. pneumoniae*.^1^

### Antimicrobial susceptibility testing

All of the clinical isolates were tested for susceptibility to 16 antimicrobial agents, including ertapenem, imipenem, and meropenem. Minimum inhibitory concentrations (MICs) of antimicrobial agents were determined using the agar dilution method of the Clinical and Laboratory Standards Institute (CLSI), while the broth dilution method was used for colistin and tigecycline (CLSI, 2019). For colistin, the breakpoints defined by the European Committee on Antimicrobial Susceptibility Testing (EUCAST)^2^ were applied, whereas the breakpoints of tigecycline were interpreted according to the US Food and Drug Administration (FDA). *E. coli* ATCC 25922 was used as a quality control strain.

### Whole-genome sequencing and bioinformatics analysis

A total of 21 CRKP isolates carrying CRISPR-Cas systems were sequenced using a HiSeq X10 Sequencer (Illumina, San Diego, CA, United States), with 150 bp paired-end short reads and 200X coverage. Of the 21 isolates, 13 isolates were also subjected to long-read sequencing using a MinION Sequencer (Nanopore; Oxford, United Kingdom). Both short and long reads were utilized to generate a *de novo* hybrid assembly using Unicycler (Wick et al., 2017) and Pilon (Walker et al., 2014). CRISPRCasFinder^3^ was used to detect CRISPR arrays (repeats and spacers) and *cas* genes in the genomes. Acquired antimicrobial resistance genes and IS were identified by ResFinder 3.2 and ISfinder (Siguier et al., 2006) using BLAST (Zankari et al., 2012). *tra* gene cluster was identified by ICEfinder (Liu et al., 2019). Replicon STs of plasmids were determined using the pMLST database.^4^ Comparisons of the sequences were plotted using BRIG (Alikhan et al., 2011).

### Statistical analysis

Fisher’s exact test was used for comparisons of the presence of CRISPR-Cas systems between the susceptible and resistant isolates. Chi-square tests were used to test the difference between carbapenem-susceptibility and CRISPR-Cas systems. Statistical significance was assessed using Prism 8 (GraphPad Prism) software. *p-*values of <0.05 were considered to be significant.

## Results

### Prevalence of CRISPR-Cas systems in clinical isolates of *Klebsiella pneumoniae*

Overall, 23.5% (164/697) of clinical *K. pneumoniae* isolates had CRISPR-Cas systems, and type I-E^*^ was more common (15.9%, 111/697) than type I-E (7.7%, 54/697). One isolate had both type I-E^*^ and type I-E systems, which has not been reported previously in *K. pneumoniae*.

**Figure 1:**
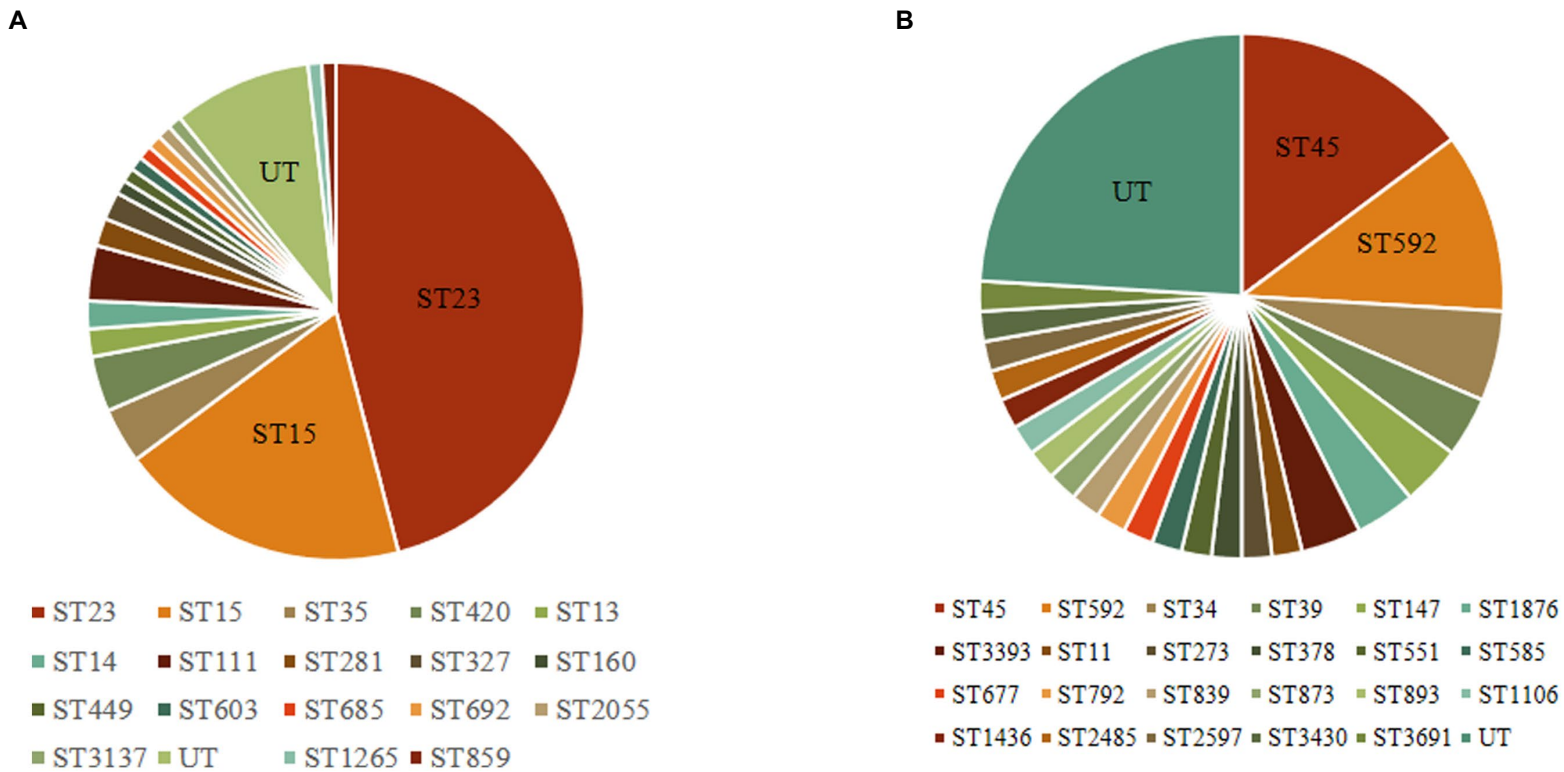
Multi-locus sequence typing of 164 clinical isolates carrying CRISPR-Cas systems. **(A)** Type I-E^*^ CRISPR systems (*n* = 111). **(B)** Type I-E CRISPR systems (*n* = 54). UT, untypable.

The 164 CRISPR-positive isolates comprised 41 different MLST types (23 isolates were untypable). Type I-E^*^ CRISPR-Cas was found in 18 STs, while type I-E CRISPR-Cas was present in 23 STs (Figure 1). The most prevalent ST among the isolates carrying type I-E^*^ CRISPR was ST23 (*n* = 51, 45.9%), followed by ST15 (*n* = 21, 18.9%). The most common ST among type I-E isolates was ST45 (14.8%), and the second was ST592 (11.1%). These results suggested that the distribution of CRISPR-Cas types was associated with particular STs among clinical isolates of *K. pneumoniae*. Among the 164 CRISPR-positive isolates, only one isolate (BJKP38) was ST11, indicating that CRISPR-Cas loci are rare in ST11 *K. pneumoniae*.

### Presence of type I-E^*^ and type I-E CRISPR-Cas systems had a negative correlation with antimicrobial resistance

The CRISPR-positive isolates (harboring either the type I-E^*^ or the type I-E CRISPR-Cas system) were significantly more susceptible to 10 antimicrobials tested, including amikacin, aztreonam, cefotaxime, cefoxitin, ceftazidime, ciprofloxacin, ertapenem, fosfomycin, imipenem, and meropenem, compared with the CRISPR-negative isolates (Table 1). The rate of carbapenem-resistance of 697 *K. pneumoniae* was 37.1% (259/697), and the rates of carbapenem-resistance of CRISPR-positive and -negative isolates were 12.8% (21/164) and 44.7% (238/533), respectively (Table 2). Of the 21 CRKP isolates, 17 carried type I-E^*^ and four carried type I-E CRISPR-Cas systems.

**Table 1:** The association of antimicrobial susceptibilities with CRISPR-Cas systems in clinical isolates of *Klebsiella pneumoniae*.

	CRISPR-Cas system [Number of susceptible isolates (%)]				
	Type I-E^*^ (*n* = 111)	Type I-E (*n* = 54)	Absent (*n* = 533)	*p-*value	
Amikacin	98 (88.3)	49 (90.7)	346 (64.9)	**<0.001**	**<0.001**
Aztreonam	72 (64.9)	39 (72.2)	208 (39.0)	**<0.001**	**<0.001**
Cefotaxime	65 (58.6)	35 (64.8)	171 (32.1)	**<0.001**	**<0.001**
Cefoxitin	83 (74.8)	35 (64.8)	232 (43.5)	**<0.001**	**<0.001**
Ceftazidime	74 (66.7)	38 (70.4)	214 (40.2)	**<0.001**	**<0.001**
Ceftazidime-avibactam	110 (99.1)	53 (98.1)	508 (95.3)	0.067	0.499
Chloramphenicol	71 (64.0)	29 (53.7)	218 (40.9)	**<0.001**	0.082
Ciprofloxacin	66 (59.5)	28 (51.9)	158 (29.6)	**<0.001**	**0.001**
Colistin	109 (98.2)	54 (100.0)	506 (94.9)	0.205	0.161
Ertapenem	96 (86.5)	47 (87.0)	278 (52.2)	**<0.001**	**<0.001**
Fosfomycin	80 (72.1)	34 (63.0)	248 (46.5)	**<0.001**	**0.021**
Imipenem	100 (90.1)	48 (88.9)	296 (55.5)	**<0.001**	**<0.001**
Meropenem	98 (88.3)	50 (92.6)	305 (57.2)	**<0.001**	**<0.001**
Minocycline	35 (31.5)	16 (29.6)	152 (28.5)	0.525	0.863
Tigecycline	95 (85.6)	45 (83.3)	432 (81.1)	0.282	0.855
Trimethoprim-sulfamethoxazole	49 (44.1)	17 (31.5)	147 (27.6)	**0.001**	0.543

*p*-values are shown as CRISPR-negative isolates compared with type I-E^*^ isolates and CRISPR-negative isolates compared with type I-E isolates. The bold *p*-values were statistically significant.

**Table 2:** The association of carbapenem-susceptibilities with CRISPR-Cas systems in clinical isolates of *Klebsiella pneumoniae*.

	CRISPR-Cas (+) *n* (%)	CRISPR-Cas (−) *n* (%)	Total	*P-*value
CRKP	21 (12.8%)	238 (44.7%)	259 (37.1%)	
CSKP	143 (87.2%)	295 (55.3%)	438 (62.8%)	
Total	164 (100%)	533 (100%)	697 (100%)	<0.001

CRKP, carbapenem-resistant *K. pneumoniae*; CSKP, carbapenem-susceptible *K. pneumoniae*.

### Characteristics of 12 ST15 and one ST23 isolates that carried both type I-E^*^ CRISPR-Cas systems and bla_KPC-2_-bearing plasmids

Although most isolates carrying CRISPR-Cas systems were susceptible to carbapenems (87.2%, 143/164), there were still 21 isolates that showed resistance to carbapenems. These 21 isolates were subjected to whole-genome sequencing and genome analysis. More than half of the isolates (*n* = 13) harbored the carbapenemase-encoding gene *bla*_KPC-2_ and type I-E^*^ CRISPR-Cas systems, and they were resistant to all three carbapenems tested. Eight other isolates, including all four isolates carrying type I-E CRISPR-Cas systems, did not produce any carbapenemase, and six of the isolates were resistant to ertapenem only, and the remaining two isolates were also resistant to the three carbapenems tested (Table 3).

**Table 3:** Characteristics of 21 CRKP clinical isolates carrying CRISPR-Cas systems.

Isolate	CRISPR type	Carbapenemase	Extended-spectrum β-lactamase	*bla*_KPC-2_-carrying plasmid size (Kb)	*bla*_KPC-2_-carrying plasmid type	ST	MIC (μg/mL)		
							ETP	IPM	MEM
HSKP5	Type I-E^*^	KPC-2	CTX-M-15, SHV-106, SHV-28	94.1	IncFII_K34_	15	>32	32	64
HSKP8	Type I-E^*^	KPC-2	CTX-M-15, SHV-106, SHV-28	94.4	IncFII_K34_	15	32	8	4
HSKP33	Type I-E^*^	KPC-2	CTX-M-3, SHV-106, SHV-28	120.5	IncFII_K34_	15	>32	64	128
HSKP43	Type I-E^*^	KPC-2	CTX-M-15, SHV-106, SHV-28	94.1	IncFII_K34_	15	>32	64	128
HSKP86	Type I-E^*^	KPC-2	CTX-M-15, SHV-106, SHV-28	94.1	IncFII_K34_	15	>32	32	128
HSKP104	Type I-E^*^	KPC-2	CTX-M-15, SHV-106, SHV-28	94.1	IncFII_K34_	15	>32	32	64
HSKP107	Type I-E^*^	KPC-2	CTX-M-15, SHV-106, SHV-28	94.1	IncFII_K34_	15	>32	32	64
RJKP14	Type I-E^*^	KPC-2	SHV-106, SHV-28	108.8	IncFII_K34_	15	>32	128	64
HSKP39	Type I-E^*^	KPC-2	SHV-12, SHV-13, SHV-31, SHV-129	114.7	IncFII_K34_	23	>32	16	>128
HSKP1	Type I-E^*^	KPC-2	SHV-106, SHV-28	142.1	IncFII(PHN7A8)	15	>32	128	128
RJKP41	Type I-E^*^	KPC-2	CTX-M-65, SHV-106, SHV-28	164.6	IncFII(PHN7A8)	15	>32	128	64
GZKP13	Type I-E^*^	KPC-2	CTX-M-14, SHV-106, SHV-28	56.2	IncFIA(HI1)	15	>32	32	64
RJKP36	Type I-E^*^	KPC-2	CTX-M-15, SHV-106, SHV-28	108.8	IncR	15	16	16	8
HSKP69	Type I-E	–	SHV-26, SHV-78, SHV-98, SHV-145	–	–	45	>32	64	>128
HSKP40	Type I-E	–	SHV-5, SHV-2, SHV-102	–	–	UT	>32	8	>128
GSKP2	Type I-E^*^	–	CTX-M-14, SHV-33	–	–	449	32	0.25	0.06
SCKP43	Type I-E^*^	–	CTX-M-55, SHV-106, SHV-28	–	–	15	16	1	0.5
ZJKP62	Type I-E^*^	–	SHV-75	–	–	420	16	0.25	0.5
HSKP140	Type I-E	–	CTX-M-3, SHV-110, SHV-191	–	–	2,485	8	0.25	1
HSKP155	Type I-E	–	SHV-27	–	–	3,393	8	0.5	0.25
ZJKP12	Type I-E^*^	–	SHV-106, SHV-28	–	–	14	4	0.5	1

ETP, ertapenem; IPM, imipenem; MEM, meropenem; MIC, minimum inhibitory concentration; ST, sequence type.

The spacer sequences of CRISPR-Cas systems in these 21 isolates were analyzed and compared the difference between the two groups (13 isolates vs. 8 isolates, Supplementary Table S2). The 13 isolates with *bla*_KPC-2_-bearing plasmids mainly carried 18 spacers, of which ten spacers were present in 12 isolates, six in 11 isolates, and two in seven isolates. The eight isolates without KPC-2 contained 166 spacers, and each one existed in one to three isolates.

MLST analysis showed that among the 13 isolates carrying both type I-E^*^ CRISPR-Cas systems and *bla*_KPC-2_, 12 belonged to ST15 and one was ST23 (Table 3), while only eight (5.6%, 8/143) isolates belonged to ST15 in carbapenem-susceptible *K. pneumoniae* carrying CRISPR-Cas systems. The 12 ST15 isolates were collected from three hospitals, including Huashan Hospital (*n* = 8) and Ruijin Hospital (*n* = 3) in Shanghai, and the First Affiliated Hospital of Guangzhou Medical University (*n* = 1). The isolation dates of the 12 isolates were from July 2018 to January 2019.

### Presence of a new type of IncFII_K34_ in nine bla_KPC-2_-carrying plasmids

**Figure 2:**
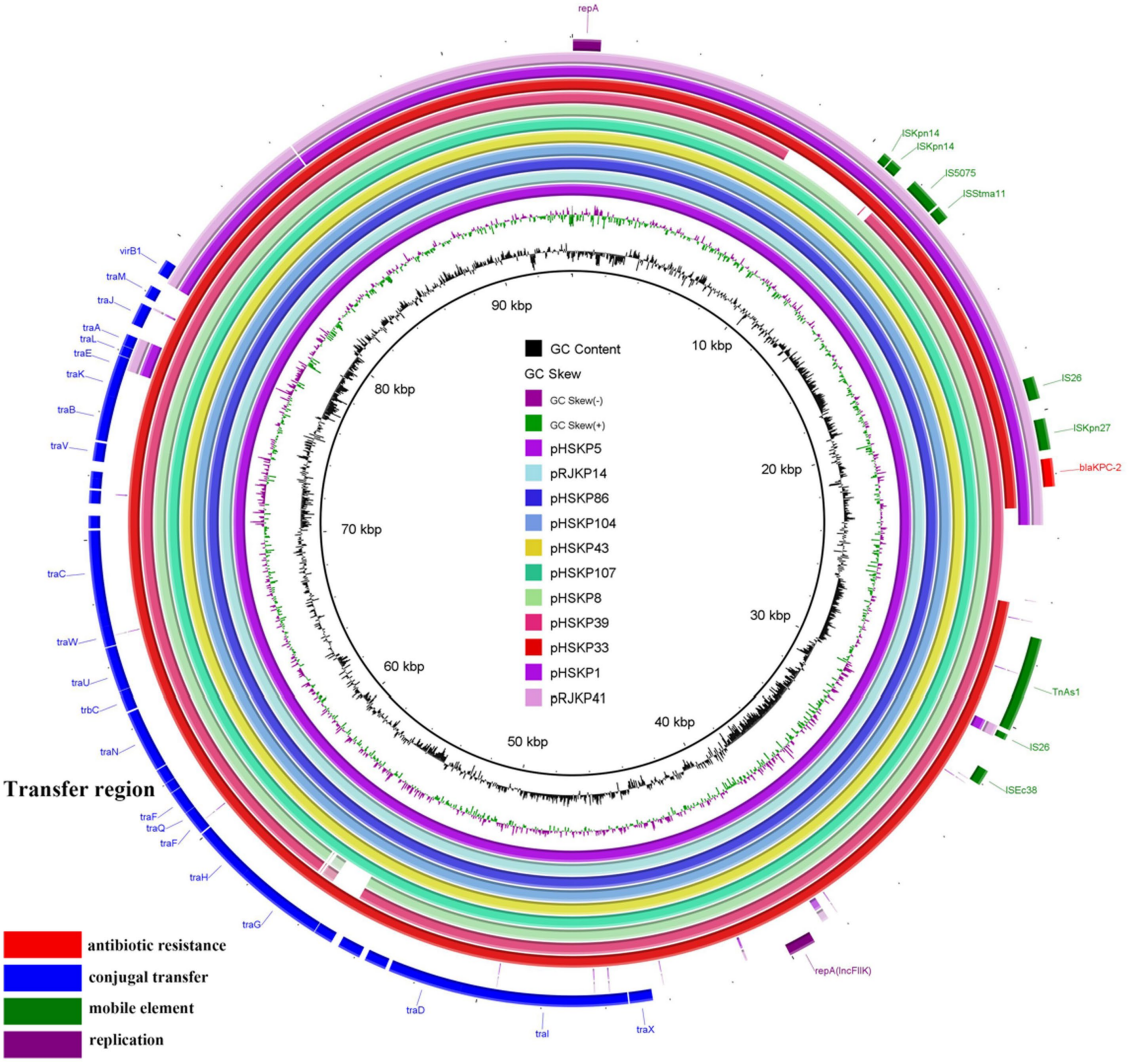
Comparative analysis of the nine IncFII_K34_ plasmids and two IncFII(PHN7A8) plasmids. The outmost purple and pink circles represent the two IncFII(PHN7A8) plasmids which showed low homology with IncFII_K34_.

The *bla*_KPC-2_ genes were located on plasmids in all 13 isolates. Of note, *bla*_KPC-2_-carrying plasmids of nine isolates belonged to IncFII_K34_ (Table 3), and the sequences of the nine plasmids were highly conserved (Figure 2). The IncFII_K34_ replicon was first identified by our group during a study on ST23 CR-HvKP in China and has been submitted to the plasmid MLST database. The IncFII_K34_ plasmid was recognized by comparing the sequence of the *copA* region of the plasmid with the other IncFII_K_ alleles in the database. All the nine IncFII_K34_ plasmids harbored *bla*_KPC-2_, meanwhile, pHSKP33 harbored *dfrA14* and *qnrS1* and pHSKP39 harbored *aac(3)-IId, bla_SHV-12_* and *bla_TEM-1B_* additionally. Besides, *tra* gene cluster that encodes proteins needed for conjugation was identified on all the IncFII_K_ plasmids, while was absent on IncFII(PHN7A8) plasmids. The *bla*_KPC-2_-carrying plasmid types of HSKP1 and RJKP41 were IncFII(PHN7A8), the most common type among ST11 KPC-producing *K. pneumoniae*, and the plasmid types of the remaining two isolates were IncFIA(HI1) and IncR.

Among the 13 isolates that carried both type I-E^*^ CRISPR-Cas systems and *bla*_KPC-2_-bearing plasmids, 11 isolates had self-targeting spacers matching their respective protospacers on *bla*_KPC-2_-bearing plasmids, of which two isolates (HSKP1 and RJKP41) had point mutations. The sequence of the self-targeting spacer was 5’-CCGCCGTTT **A** ATCGCGGTGATGATATCCGGCA-3′ (the point mutation is shown in bold and underlined). The remaining two isolates (GZKP13 and HSKP39) had no spacer matching their *bla*_KPC-2_ plasmids. The sequences of *hns* gene encoding transcriptional repressor H-NS, which showed to prevent the plasmids transformation (Lin et al., 2016), from 13 isolates were identical to that from *K. pneumoniae* NTUH-K2044. In addition, no mutation of CRISPR-Cas loci was identified on 13 genomes compared to these sequences from CRISPR-Cas finder database.

## Discussion

The prevalence of the CRISPR-Cas system in *K. pneumoniae* was reported to vary from 12.4 to 30.7% (Lin et al., 2016; Li et al., 2018; Wang et al., 2020). Consistently, we found that the CRISPR-Cas system was positive in 164/697 (23.5%) of *K. pneumoniae* isolates collected from 11 hospitals in China. Previous studies have demonstrated that the scarcity of type I-E CRISPR-Cas systems in the CC258 lineage (including ST11 and ST258) allowed them to readily acquire and adapt to *bla*_KPC_-plasmids, and in our study, only one isolate was ST11 among CRISPR-positive *K. pneumoniae*. It is also notable that ST23, a common ST of the hyper-virulent *K. pneumoniae*, was the most prevalent ST among type I-E^*^ CRISPR-positive isolates. In this study, one CRISPR-positive ST23 isolate also carried a *bla*_KPC-2_-plasmid.

This work showed that nearly all the CRKP isolates carrying both type I-E^*^ CRISPR-Cas system and *bla*_KPC-2_-bearing plasmids belonged to ST15. ST15 isolates of *K. pneumoniae* are emerging international MDR clones, and the molecular features of ST15 are relatively unknown. In this study, ST15 was one of the most prevalent ST carrying type I-E^*^ CRISPR-Cas systems. Taken together, it appears that type I-E^*^ CRISPR-Cas systems could not prevent the entrance of resistant plasmids into ST15 isolates. Moreover, *bla*_KPC-2_-carrying plasmid types of CRKP carrying type I-E^*^ CRISPR-Cas systems were mainly the new IncFII_K34_, and the sequences of these plasmids are highly conserved.

Most isolates had self-targeting spacers matching their plasmids, only two isolates had point mutations. A previous study demonstrated that single or multiple mutations within the protospacer, but outside a seven-nucleotide seed region immediately following the essential protospacer-adjacent motif, do not lead to the escape of exogenous nucleic acid of viruses (Semenova et al., 2011). It has been reported that anti-CRISPR proteins could inhibit the function of CRISPR-Cas systems (Pawluk et al., 2014), and therefore whether anti-CRISPR proteins existed in these isolates remained to be further studied. In addition, a previous study indicated that CRISPR-Cas immunity is not absolute. It reduces the rate of receipt of the plasmid, but does not prevent its transfer and establishment (Jiang et al., 2013). Hence, further studies are needed to explore the mechanisms by which the IncFII_K34_ plasmids evaded type I-E^*^ CRISPR-Cas immunity in *K. pneumoniae*.

CRISPR-Cas systems are associated with antimicrobial susceptibility in *Streptococcus pyogenes*, *Pseudomonas aeruginosa*, *Escherichia coli*, and *K. pneumoniae* (Zheng et al., 2014; van Belkum et al., 2015; Aydin et al., 2017; Wang et al., 2020). A previous study showed an inverse correlation between the presence of the type I-E^*^ CRISPR-Cas system and antimicrobial resistance, but did not show an association between the distribution of type I-E CRISPR and antimicrobial susceptibilities in *K. pneumoniae* (Li et al., 2018). Our data showed that the absence of the type I-E^*^ and type I-E CRISPR-Cas systems both contributed to the acquired antimicrobial resistance in *K. pneumoniae*.

In this study, eight CRKP isolates did not produce any carbapenemases, and six of them were resistant to ertapenem only, but were susceptible to meropenem and imipenem. A previous study of our research group demonstrated that mutations in *ramR* caused the over-expression of efflux pump and the inhibition of outer membrane protein OmpK35, which was implicated in ertapenem resistance only in *K. pneumoniae* including the six isolates in this study (Wang D. et al., 2022).

In summary, 23.5% (164/697) of clinical *K. pneumoniae* isolates had type I-E^*^ or type I-E CRISPR-Cas systems in samples collected in China. Isolates carrying the CRISPR-Cas system were more susceptible to carbapenems, compared with CRISPR-negative isolates. IncFII plasmids could co-exist with the type I-E^*^ CRISPR-Cas systems in ST15 *K. pneumoniae*, despite the CRISPR-Cas systems contained a spacer matching their own KPC-2 plasmids.

## Data availability statement

Publicly available datasets were analyzed in this study. This data can be found at: DDBJ/ENA/GenBank under the bioproject PRJNA8534.

## Author contributions

MW and QG designed the research and edited the manuscript. YH performed the experiments and wrote the manuscript. JJ did the molecular data analysis and wrote the manuscript. DW performed the experiments. All authors contributed to the article and approved the submitted version.

## Funding

This work was supported by the National Natural Science Foundation of China (grant number 81991531).

## Conflict of interest

The authors declare that the research was conducted in the absence of any commercial or financial relationships that could be construed as a potential conflict of interest.

## Publisher’s note

All claims expressed in this article are solely those of the authors and do not necessarily represent those of their affiliated organizations, or those of the publisher, the editors and the reviewers. Any product that may be evaluated in this article, or claim that may be made by its manufacturer, is not guaranteed or endorsed by the publisher.

## Footnotes

^1^ http://bigsdb.pasteur.fr/klebsiella/klebsiella.html

^2^ http://www.eucast.org/

^3^ https://crisprcas.i2bc.paris-saclay.fr/CrisprCasFinder/Index

^4^ https://cge.cbs.dtu.dk/services/pMLST/
