# Supplementary material for: Coexistence of blaKPC-IncFII plasmids and type I-E* CRISPR-Cas systems in ST15 Klebsiella pneumoniae
Source: Front Microbiol. 2023 Mar 8;14:1125531. doi: 10.3389/fmicb.2023.1125531 (PMC10030501; doi:10.3389/fmicb.2023.1125531)
Supplement: Supplementary file 1 [file Table_1.DOCX]

**Table S1.** The distribution of CRISPR-Cas systems in *Klebsiella pneumoniae* clinical isolates among 11 hospitals

| **Hospital** | **Province** | **Isolates** | **CRISPR-Cas systems** | | |
| --- | --- | --- | --- | --- | --- |
|  |  |  | **Type I-E** | **Type I-E*** | **Total** |
| Huashan Hospital Affiliated to Fudan University | Shanghai | 213 | 21(9.86%) | 26(12.21%) | 47(22.07%) |
| Ruijin Hospital Affiliated to Shanghai Jiao Tong University | Shanghai | 55 | 2(3.64%) | 9(16.36%) | 11(20%) |
| Children’s Hospital Affiliated to Shanghai Jiao Tong University | Shanghai | 55 | 4(7.27%) | 3(5.45%) | 7(12.73%) |
| Sir Run Run Shaw Hospital Affiliated to Zhejiang University | Zhejiang | 64 | 4(6.25%) | 9(14.06) | 13(20.31%) |
| People's Hospital of Peking University | Beijing | 45 | 6(13.33%) | 7(15.56%) | 13(28.89%) |
| Beijing Hospital of Health Ministry | Beijing | 46 | 3(6.52%) | 2(4.35%) | 5(10.87%) |
| the First Affiliated Hospital of Guangzhou Medical University | Guangdong | 43 | 3(6.98%) | 12(27.91%) | 15(34.88%) |
| Gansu Provincial People's Hospital | Gansu | 37 | 6(16.22%) | 11(29.73%) | 16(43.24%)^*^ |
| the First Affiliated Hospital of Kunming Medical University | Yunnan | 44 | 0(0%) | 9(20.45%) | 9(20.45%) |
| Sichuan Provincial People's Hospital | Sichuan | 48 | 3(6.25%) | 9(18.75%) | 12(25%) |
| China-Japan Union Hospital of Jilin University | Jilin | 47 | 2(4.26%) | 14(29.79%) | 16(34.04%) |
| Total |  | 697 | 54(7.75%) | 111(15.93%) | 164(23.53%)^*^ |

^*^One isolate that had both Type I-E and Type I-E^*^ CRISPR systems.
